# Dual-targeting tigecycline nanoparticles for treating intracranial infections caused by multidrug-resistant *Acinetobacter baumannii*

**DOI:** 10.1186/s12951-024-02373-z

**Published:** 2024-03-30

**Authors:** Xing Lan, Shugang Qin, Huan Liu, Mengran Guo, Yupei Zhang, Xinyang Jin, Xing Duan, Min Sun, Zhenjun Liu, Wenyan Wang, Qian Zheng, Xuelian Liao, Jinpeng Chen, Yan Kang, Yongmei Xie, Xiangrong Song

**Affiliations:** 1 Department of Critical Care Medicine, Department of Clinical Pharmacy, Frontiers Science Center for Disease-Related Molecular Network, State Key Laboratory of Biotherapy and Cancer Center, West China School of Nursing, West China Hospital, Sichuan University, Chengdu, China; 2 State Key Laboratory of Drug Delivery and Pharmacokinetics, Tianjin Institute of Pharmaceutical Research, Tianjin, 300301 People’s Republic of China; 3 School of Pharmacy, Faculty of Medicine, Macau University of Science and Technology, Macau, China; 4 Shihezi University, Xinjiang, China

**Keywords:** Nanoparticles, Blood–brain barrier, Multidrug-resistant *Acinetobacter baumannii*, Tigecycline, Intracranial infection

## Abstract

Multidrug-resistant (MDR) Acinetobacter baumannii (*A. baumannii*) is a formidable pathogen responsible for severe intracranial infections post-craniotomy, exhibiting a mortality rate as high as 71%. Tigecycline (TGC), a broad-spectrum antibiotic, emerged as a potential therapeutic agent for MDR *A. baumannii* infections. Nonetheless, its clinical application was hindered by a short in vivo half-life and limited permeability through the blood–brain barrier (BBB). In this study, we prepared a novel core–shell nanoparticle encapsulating water-soluble tigecycline using a blend of mPEG-PLGA and PLGA materials. This nanoparticle, modified with a dual-targeting peptide Aβ11 and Tween 80 (Aβ11/T80@CSs), was specifically designed to enhance the delivery of tigecycline to the brain for treating *A. baumannii*-induced intracranial infections. Our findings demonstrated that Aβ11/T80@CSs nanocarriers successfully traversed the BBB and effectively delivered TGC into the cerebrospinal fluid (CSF), leading to a significant therapeutic response in a model of MDR *A. baumannii* intracranial infection. This study offers initial evidence and a platform for the application of brain-targeted nanocarrier delivery systems, showcasing their potential in administering water-soluble anti-infection drugs for intracranial infection treatments, and suggesting promising avenues for clinical translation.

**Graphical abstract:**

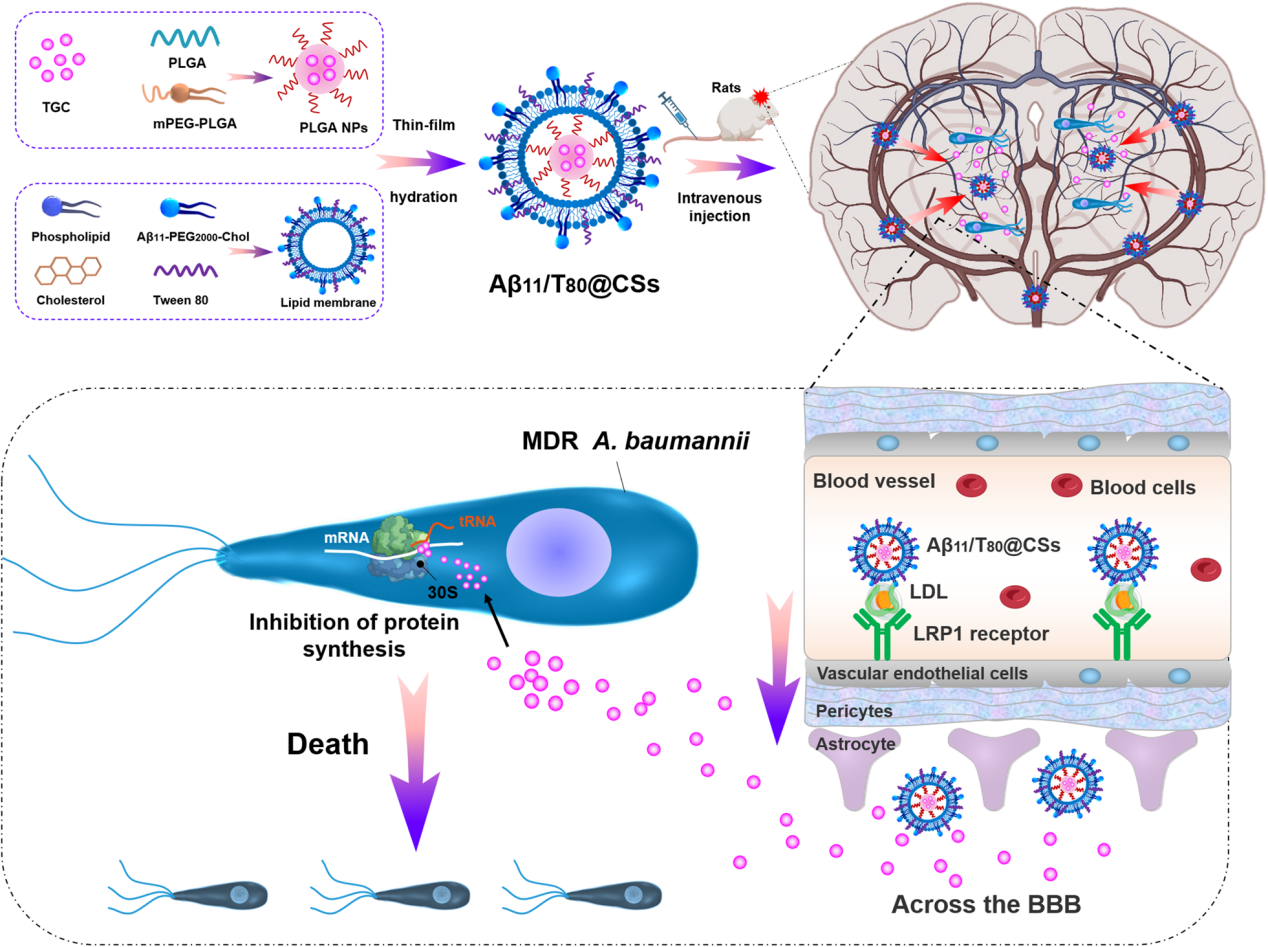

**Supplementary Information:**

The online version contains supplementary material available at 10.1186/s12951-024-02373-z.

## Introduction

*Acinetobacter baumannii* (*A. baumannii*), a notorious nosocomial pathogen, was identified as a primary cause of severe intracranial infections and associated complications [1]. Recent data indicated a rising trend in the incidence of postoperative intracranial infections attributable to *A. baumannii*, accounting for 15.7–24.2% of cases [2, 3]. The proliferation of multidrug resistance (MDR) in *A. baumannii*, fueled by antibiotic misuse, rendered the treatment of such infections exceedingly challenging [4]. Reports highlighted that MDR *A. baumannii* showed resistance to multiple antibiotic classes, including β-lactams, cephalosporins, and carbapenems, with the MDR rate escalating from 23 to 63%, a rate fourfold higher than that observed in other MDR Gram-negative bacteria like *Pseudomonas aeruginosa* and *Klebsiella pneumoniae* [5].

Tigecycline (TGC), a new-generation tetracycline antibiotic, emerged as the most potent treatment against intracranial infections caused by MDR *A. baumannii* [6]. TGC functioned by binding to the 30S ribosomal subunit, thereby inhibiting bacterial protein synthesis through prevention of tRNA binding at the ribosomal A site, ultimately stalling bacterial growth [7, 8]. Notably, TGC retained efficacy against MDR strains by circumventing resistance mechanisms such as ribosomal protection and antibiotic efflux, a significant advantage over minocycline [9, 10]. However, TGC’s limited permeability through the blood–brain barrier (BBB) posed challenges in attaining adequate drug concentrations in the cerebrospinal fluid (CSF). Consequently, the development of a safe and effective TGC brain-targeted delivery system was deemed essential.

Poly (lactic-co-glycolic acid)-poly (ethylene glycol) (PLGA-PEG) nanoparticles can traverse the blood–brain barrier (BBB) through multiple mechanisms, including passive targeting (via small size and PEGylated surface properties), intracellular penetration (such as endocytosis) and active targeting (by modifying ligands with specific affinity) [11]. This approach offered an innovative strategy for transporting drugs across the blood–brain barrier (BBB), yet reports on targeted therapy for intracranial infections remained sparse [12]. β-Amyloid (Aβ1-40), a peptide resulting from the proteolytic cleavage of the amyloid precursor protein, circulated in blood, cerebrospinal fluid (CSF), and interstitial fluid. Aβ1-40 can cross the BBB by binding to low-density lipoproteins (LDL, such as ApoE and ApoA) and LDL receptor-related protein 1 (LRP1) [13–15]. A fragment of Aβ1-40, Aβ25-35 (Aβ11), exhibits similar functionality [16]. Tween 80 (T80), a nonionic surfactant, was observed to enhance the accumulation of nanoparticles in brain endothelial cells of the BBB [12]. Moreover, nanoparticles modified with T80 were reported to adsorb LDL in blood and be uptaken by BBB endothelial cells via interaction with LRP1 [17]. Additionally, T80 was found to inhibit the active efflux of P-glycoprotein, increasing the brain uptake of nanoparticles [18]. These findings suggested that the Aβ11 and T80 dual-modified nano-delivery system held significant potential for the brain-targeted delivery of Tigecycline (TGC) to treat intracranial infections.

In this study, we introduced a pioneering method to prepare core–shell nanoparticles modified with Aβ11 and Tween 80 (Aβ11/T80@CSs) for delivering water-soluble TGC into the CSF by traversing the BBB. We discovered that the Aβ11/T80@CSs brain-targeting nano-delivery system could enhance the encapsulation efficiency of water-soluble drugs, prolong TGC's in vivo circulation time, and improve its bioavailability. The Aβ11/T80@CSs system facilitated TGC distribution in the brain and augmented its efficacy against MDR *A. baumannii* in a mouse model of intracranial infection. Our findings provided a foundational framework for further development of brain-targeting nano-delivery systems in the treatment of intracranial infections caused by MDR bacteria.

## Experimental section

### Materials

Tigecycline, with a purity exceeding 98%, was procured from Suo Laibao Biotechnology Co., Ltd. The Aβ11 peptide, featuring an additional cysteine at its C-terminus (Aβ11-Cys), was synthesized by Qiang Yao Biochem Ltd., Hubei, China. The C-terminal amidated Aβ11-Cys peptide sequence was NH_2_-CGSNKGAIIGLM-CONH_2_. Mal-PEG2000-Chol was acquired from Pengshuo Biotechnology Co. Ltd., Chengdu, China. Various forms of mPEG-PLGA, PLGA, and PLGA-PEG-PLGA, all with a molecular weight of 15 kDa and varying lactide to glycolide ratios, were purchased from Jinan Daigang Biomaterial Co., Ltd., Jinan, China. Poly (vinyl alcohol) (PVA) with a molecular weight range of 30–70 kDa, Tween 80, and 3-[4,5-dimethylthiazole-2-yl]-2,5-diphenyltetrazolium bromide (MTT) were obtained from Chengdu Real Biotechnology Co., Ltd., Chengdu, China. 1,1'-Dioctadecyl-3,3,3',3'-Tetramethylindodicarbocyanine perchlorate (DiD) was sourced from Gelman Sciences Inc., California, USA. Confocal plates, fetal bovine serum and DMEM (Biosharp) were procured from Rancho Technology Co., Ltd., Beijing, China. All other reagents and chemicals were of analytical reagent grade and were used without further purification.

### Animals

BALB/c mice, male, aged 6–8 weeks and weighing 22–25 g (SPF grade), along with adult healthy female Sprague–Dawley rats, weighing between 200 and 250 g (SPF grade), were obtained from the Sichuan University Experimental Animal Center, Ltd., Chengdu, Sichuan, China. All animal experiments were conducted with the approval and under the supervision of the West China Hospital Animal Care and Use Committee, Sichuan University (20220422002). To minimize discomfort for animals in our study, we employed isoflurane gas anesthesia during the model preparation phase for rats, ensuring that the duration of exposure did not exceed 30 s for each animal. Following bacterial infection, rats were housed in clean cages, under the care of a dedicated handler. Bedding was replaced every 3 days, and fresh water and feed were replenished every 2 days. Euthanasia was conducted through cervical dislocation after inducing anesthesia with isoflurane. The animals were housed in an environment maintained at 22 ± 1 °C with a 12:12 h light–dark cycle and a relative humidity of 55 ± 10%.

### Synthesis of Chol-PEG2000-Aβ11

Chol-PEG2000-Aβ11 was synthesized via a Michael addition reaction. Initially, 50 mg of Chol-PEG2000-Mal was dissolved in 5 mL of chloroform and subsequently transformed into a film through rotary evaporation. This film was then completely dissolved by hydrating with 3 mL of Milli-Q water for 30 min. In a separate procedure, 22 mg of thiodized Aβ11-Cys was dissolved in 2 mL of Milli-Q water and combined with the Chol-PEG2000-Mal suspension. Following this, 200 μL of EDTA (500 mM, pH 8.0) and 3 mL of 0.1 M phosphate buffer were added to the mixture. The reaction proceeded at room temperature for 48 h. Unbound peptide and Chol-PEG2000-Mal were removed by dialysis (MW = 5 kDa) over a period of 72 h, after which Chol-PEG2000-Aβ11 was obtained through freeze-drying.

### Formulation screening of PLGA nanoparticles

To select the optimal polymer carrier material for encapsulating Tigecycline (TGC), we synthesized seven different formulations of PLGA nanoparticles (PLGA NPs), detailed in Table 1. These PLGA NPs were prepared using an emulsification-solvent evaporation method. Briefly, PLGA was dissolved in a mixture of dichloromethane (DCM) and acetone to create a 2% (w/v) solution, serving as the organic phase. An aqueous solution of tigecycline was then gradually added dropwise to this organic phase and emulsified using an ultrasonic liquid processor (XinZhi, China) at 100 W power, forming an oil-in-water (O/W) emulsion. This O/W emulsion was subsequently dropped into a 1% polyvinyl alcohol (PVA) solution, and a secondary ultrasound process generated a multi emulsion (W/O/W). The resultant emulsion was then transferred to a 250 mL eggplant-shaped flask, and the organic solvent was evaporated under vacuum at 37 °C, resulting in the formation of PLGA NPs. Using a similar preparation method, DiD was substituted for TGC to synthesize PLGA NPs/DiD.

**Table 1:** Polymer material composition of each formulation

Formulation	mPEG-PLGA (75/25)	PLGA (50/50)	PLGA (75/25)	PLGA-PEG-PLGA (50/50)	PLGA-PEG-PLGA (75/25)
F1	20 mg	–	–	–	–
F2	–	20 mg	–	–	–
F3	–	–	20 mg	–	–
F4	–	–	–	20 mg	–
F5	–	–	–	–	20 mg
F6	10 mg	10 mg	–	–	–
F7	10 mg	–	10 mg	–	–

### Particle size and ζ-potential measurement

The particle size and ζ-potential of the nanoparticles were assessed using a Malvern particle size meter (Zetasizer NanoZS 90). Prior to measurement, the samples were diluted tenfold, and each sample underwent three separate measurements to ensure accuracy.

### TGC content and encapsulation efficiency determination

High-Performance Liquid Chromatography (HPLC) was employed to detect the Tigecycline (TGC) content and to ascertain the encapsulation efficiency of the nanoparticles. Take 100 μL of the prepared formulation and add it to 10 times its volume of methanol. Subject the mixture to ultrasonication for emulsion breaking, which is then used for the quantification of the total drug content in the formulation. To separate the nanoparticle formulation from free drug, transfer the prepared nanosuspension into an ultrafiltration tube (with a molecular weight cutoff of 3.5 kDa) and centrifuge at 3500 rpm for 15 min. The free tigecycline (TGC) will be located below the ultrafiltration tube. Collect 100 μL of the lower layer containing the free drug, dilute it tenfold with methanol, and prepare it for analysis. Chromatographic separation was conducted on a C18 column (250 mm × 4.6 mm, 6 μm) using a mobile phase composed of ammonium phosphate dibasic-triethylamine-methanol (50:1:49, pH = 6.3), at a flow rate of 1.0 mL/min. The detection wavelength was set at 246 nm. The drug loading and encapsulation efficiency (%) of the nanoparticles were calculated using the following equation: Encapsulation efficiency = $\frac{{M}_{1}-{M}_{2}}{{M}_{1}}$×100%, where M_1 represents the total weight of the drug and M_2 the weight of the drug (TGC) remaining in the liquid medium post-encapsulation.

### Preparation and characterization of the Aβ11/T80@CSs

Core–shell nanoparticles were synthesized through the thin-film hydration method [19], and the formulation with varying Aβ11 molar ratios is detailed in Table 2. Phospholipid (PLS100), cholesterol (Chol), Chol-PEG2000-Aβ11, and Chol-PEG2000 were dissolved in 4 mL of chloroform. Subsequently, the organic solvent was evaporated using a rotary evaporator. The resultant lipid membrane was hydrated with a PLGA NPs solution at 37 °C for 30 min, then sonicated at 80 W for 6 min to form lipid nanoparticles. These nanoparticles were collected in ultrafiltration tubes, centrifuged at 4000 rpm for 15 min, and washed thrice with Milli-Q water to remove unencapsulated drugs (Aβ11@CSs). Following a similar method, Tween 80 was added to the chloroform to synthesize Aβ11- and Tween 80-modified lipid nanoparticles (Aβ11/T80@CSs). Nanoparticles labeled with DiD or Cou6 were prepared by incorporating appropriate amounts of DiD or Cou6.

**Table 2:** Formulations of Aβ11 delivery systems with different molar ratios

	CSs	1%Aβ11	5%Aβ11	10%Aβ11
PLS100	60	60	60	60
Chol	30	30	30	30
PEG2000-Chol	10	9	5	0
Aβ11-PEG2000-Chol	0	1	5	10

The particle size, ζ-potential, encapsulation efficiency (EE) and morphological of the Aβ11/T80@CSs nanoparticles were measured as described previously [20–22].

### Drug release study

An in vitro drug release study was conducted employing the dialysis method, with phosphate-buffered saline (PBS, pH = 7.4) as the release medium. For this study, 2 mL aliquots of free Tigecycline (TGC), CSs, or Aβ11/T80@CSs (each containing 1 mg of TGC) were sealed in dialysis bags with a molecular weight cutoff of 3.5 kDa. These bags were then immersed in 30 mL of PBS and gently agitated at 37 °C. At predetermined time intervals, 1.0 mL of PBS was sampled from outside each dialysis bag for HPLC analysis, and an equivalent volume of fresh PBS was replenished to maintain a constant volume in the release medium.

### Brain distribution imaging experiment

BALB/c mice underwent a one-week acclimation period to their new environment. Subsequently, they were randomly assigned to different groups. The mice received injections of nanoparticles or free DiD (equivalent to 0.5 mg/kg DiD) via the tail vein. A blank control group, comprising three mice, was also established. Four hours post-injection, the mice were euthanized using isoflurane, and their brains were harvested for in vitro imaging. The imaging was performed using the IVIS imaging system (IVIS Lumina III, PerkinElmer, USA), with the excitation and emission wavelengths set at 720 nm and 740 nm, respectively.

### Cell uptake

The uptake of Cou6-loaded nanoparticles by cells was investigated using bEnd.3 endothelial cells. bEnd.3 cells, seeded at a density of 1 × 10^5^ cells/well, were cultured in 24-well plates at 5%CO_2_, 37 ℃for 24 h until they reached full adherence. The cells were subsequently incubated with CSs-Cou6, Aβ11@CSs-Cou6, or Aβ11/T80@CSs-Cou6 in confocal dishes for four hours. Post-incubation, the cells were stained with DAPI for 20 min and imaged using a laser-scanning microscope (CLSM, LSM-880). Fluorescence intensity was quantified using ImageJ software (java8, NIH, USA) and flow cytometry analysis (2060R, NovoCyte, ACEA, USA).

### Transmembrane transport assay

An in vitro blood–brain barrier (BBB) model using bEnd.3 cells was developed to assess nanoparticle penetration. 2.0 × 10^5^ cells were cultured in a Transwell chamber, achieving an intercellular compactness verified at 200 Ω·cm^2^ transendothelial electrical resistance. The model assessed penetration of TGC, CSs-TGC, Aβ11@CSs-TGC, and Aβ11/T80@CSs-TGC through the monolayer. Using Hank's Balanced Salt Solution, nanoparticles were added to the apical side and co-incubated. At intervals, 200 μL HBSS was sampled from the basolateral side for TGC concentration analysis via HPLC. Cumulative TGC permeation and apparent permeability coefficient (Papp) were calculated using the formula:

$${\text{Mn}}\, = \,C_{n} \, \times \,V\, + \,\mathop \sum \limits_{i = 1}^{n - 1} C_{i} \, \times \,V_{i}$$

$${\text{Papp}}\, = \,\frac{dQ}{{dt}}\, \times \,\frac{1}{{A\, \times \,C_{0} }}\, \times \,{1}00\%$$

where Mn represents the cumulative amount of TGC permeated at the nth time point, Cn is the concentration of TGC at the nth time point, V is the total volume of the basolateral solution, Ci is the concentration of TGC at the ith time point, and Vi is the volume of the sample collected at the ith time point. The Papp was calculated as dQ/dt × 1/(A × C0) × 100%, where dQ/dt is the rate of TGC transfer from the upper to the lower layer of the Transwell plate,* C*_*0*_ is the initial concentration of TGC in the upper layer, and A is the surface area of the membrane (*cm2*) [23].

### Antimicrobial activity of Aβ11/T80@CSs in vitro and in CSF

To evaluate the antibacterial effect of Aβ11/T80@CSs-TGC on multidrug-resistant *A. baumannii*, the minimum inhibitory concentrations were determined. Mid-log phase bacteria, diluted to 1 × 10^5^ CFU/mL, were mixed with these compounds (0.125–16 μg/mL) and incubated in 96-well plates for 24 h, with bacterial density measured at 600 nm. In a rat model, MDR *A. baumannii* was injected intracisternally (20 μL, 1 × 10^8^ CFU/mL) into the CSF. Infected rats received intrathecal injections of Aβ11/T80@CSs-TGC, CSs-TGC, or free TGC (10 μg TGC equivalent). CSF was collected after 24 h and cultured, with colony counts noted after 24 h at 37 °C.

### Pharmacokinetics of Aβ11/T80@CSs

In the pharmacokinetic study, adult female Sprague–Dawley rats, weighing between 200 and 250 g, were used. Prior to the experiment, the animals were fasted overnight with free access to water. Nine rats were randomly divided into three groups: free-TGC, CSs-TGC, and Aβ11/T80@CSs-TGC. Each group received an intravenous administration of TGC at a dose of 12.5 mg/kg via the tail vein. Blood samples were collected at predetermined time points (0.25, 0.5, 1, 2, 4, 6, and 8 h), and then placed into 2 mL Eppendorf tubes. These samples were centrifuged at 10,000 rpm for 10 min at 4 °C to separate the plasma. A portion of the plasma was mixed with methanol in a 1:4 volume ratio and further centrifuged at 13,000 rpm for 10 min at 4 °C. The supernatant was collected for TGC concentration determination using HPLC. Pharmacokinetic parameters were subsequently calculated using the statistical analysis tool DAS2.

### Anti-infective efficacy of Aβ11/T80@CSs in vivo

Rats with intracranial infections were randomly divided into four groups to evaluate the anti-infective efficacy of different treatments. These groups received saline, free Tigecycline (TGC), CSs-TGC, or Aβ11/T80@CSs-TGC (each with a TGC dose of 30 mg/kg) as treatments. The administrations were conducted intravenously at 0 h, 6 h, and 18 h post-infection modeling. After 24 h, cerebrospinal fluid (CSF) samples were collected from the rats, evenly spread onto solid culture media, and incubated at 37 °C for 24 h. The colony count for each group was then recorded.

### Hemolysis of Aβ11/T80@CSs

The hemolytic activity of Aβ11/T80@CSs was assessed using erythrocytes from healthy rats. Blood samples were collected, and red blood cells were isolated through centrifugation, followed by suspension in normal saline to create a 2% erythrocyte solution. Two milliliters of this erythrocyte suspension were mixed with free TGC, CSs, or Aβ11/T80@CSs and incubated at 37 °C for three hours. Post-incubation, the samples were centrifuged, and the absorbance of the supernatant was measured using spectrophotometry at 570 nm. Purified water and normal saline served as positive and negative controls, respectively. The percentage of hemolysis was calculated using the following formula:

$${\text{Hemolysis}}\,\left( \% \right)\, = \,\frac{Asample\, - \,Anegative}{{Apositive\, - \,Anegativ}}\, \times \,100\%$$

### Safety assessment

Cell cytotoxicity was evaluated using the MTT assay in Aβ11/T80@CSs-TGC. To further investigate the biosafety of the various formulations in vivo, blood samples were collected post-administration for biochemical analysis. After euthanizing the rats, their heart, liver, spleen, lung, and kidney tissues were harvested. These tissues underwent hematoxylin and eosin (H&E) staining and were subsequently imaged using a pathology slide scanner.

### Statistical analysis

Each experimental condition was replicated in at least three parallel experiments. The results are presented as the mean ± standard deviation. GraphPad software was employed for all statistical analyses.

## Results and discussion

### Preparation of the Aβ11/T80@CSs nanodrug delivery system

**Fig. 1:**
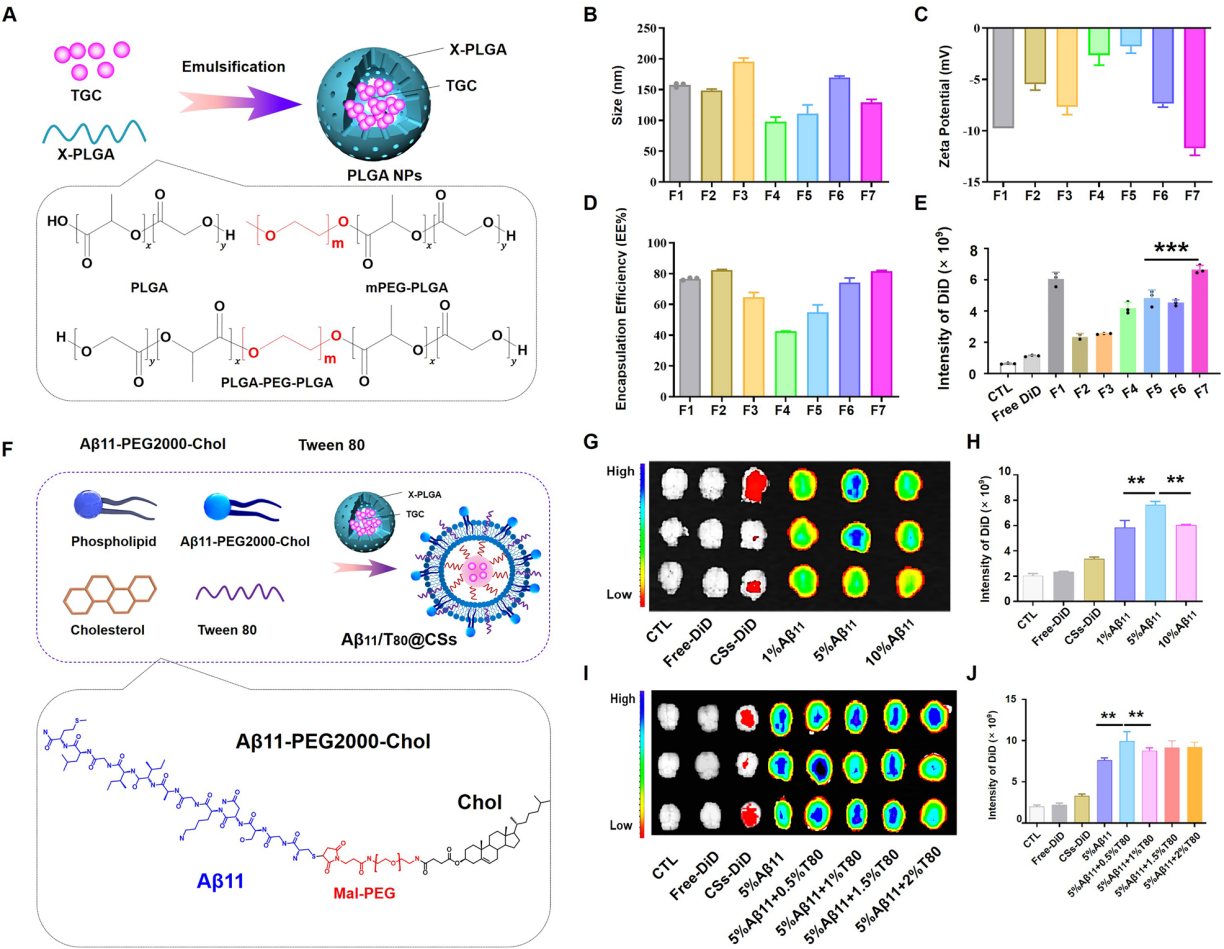
Prescription Optimization of Aβ11/T80@CSs. **A** Schematic diagram illustrating the preparation of PLGA nanoparticles (NPs). This figure shows the particle size (**B**), zeta potential (**C**), encapsulation efficiency (D), and brain distribution (**E**) of PLGA NPs synthesized from various polymer materials. **F** Schematic diagram depicting the preparation process of Aβ11/T80@CSs. The brain distribution of core–shell nanoparticles modified with varying concentrations of Aβ11 was evaluated (**G**) and quantified (**H**). Similarly, the brain distribution of core–shell nanoparticles modified with different amounts of Tween 80 was assessed (**I**) and quantified (**J**). All data are expressed as the mean ± standard deviation (SD), with n = 3 independent experiments. Statistical analysis was conducted using a t-test for panels **E**, **H**, and **J**, with significance levels indicated as follows: *p < 0.1, **p < 0.01, ***p < 0.001, ****p < *0.0001*

To optimize the formulation of poly (lactic-co-glycolic acid) nanoparticles (PLGA NPs), these nanoparticles were prepared using the emulsification-solvent evaporation method, employing PLGA series polymers as carrier materials (as illustrated in Fig. 1A). The particle size, electric potential, and encapsulation efficiency (EE%) of the PLGA NPs were characterized using Malvern particle size analyzers and High-Performance Liquid Chromatography (HPLC). The seven nanoparticle formulations exhibited a particle size range of 100–200 nm and demonstrated uniform size distribution (Fig. 1B). All formulations displayed negative zeta potentials, with formulation F7 showing the lowest zeta potential (Fig. 1C), which contributed to enhanced nanoparticle stability and reduced aggregation. With a Tigecycline (TGC) content of 3 mg, the EE% of these nanoparticles varied from 40 to 80%. Notably, formulations F2 and F7 achieved higher EE%, approximately 80%, compared to the other five formulations (Fig. 1D). The particle sizes of F4 and F5 were smaller than those of the other groups, yet their encapsulation efficiencies were significantly lower, suggesting inadequate drug encapsulation and consequently smaller empty nanoparticles.

In vivo studies were also conducted to assess variations in brain targeting among these formulations. As indicated in Fig. 1E, PLGA NPs demonstrated higher brain expression levels compared to free-DiD, implying enhanced drug delivery to the brain by the nanoparticles. The ability of the seven formulations to penetrate the blood–brain barrier (BBB) varied, with formulations F1 and F7 demonstrating stronger fluorescence absorption compared to the other formulations. It's pertinent to note the distinction in hydrophilicity between mPEG-PLGA (F1) and PLGA. The hydrophilicity of mPEG-PLGA is superior to that of PLGA, making it more suitable for encapsulating lipophilic drugs [24]. Conversely, PLGA is more frequently employed for encapsulating hydrophilic drugs [25]. Therefore, we selected formulation F7 (Containing mPEG-PLGA and PLGA) for encapsulating the water-soluble drug tigecycline (TGC). This variation in nanoparticle distribution could be attributed to the influence of the material composition on particle size and potential. PLGA polymers vary in lactic acid to glycolic acid (LA/GA) ratios, with a lower LA/GA ratio indicating increased hydrophilicity and higher zeta potential. Additionally, polyethylene glycol (PEG) modification of PLGA can further enhance its hydrophilicity, influencing nanoparticle size and encapsulation efficiency. Particle size is a critical attribute for nanoparticle characterization and a significant parameter in drug delivery systems, as it affects cellular and tissue uptake [26]. Smaller nanoparticles are generally more efficiently absorbed by cells. Recent studies have also suggested that endothelial cells preferentially uptake polymer nanoparticles with more negative charges [27]. Based on these results, F7 PLGA NPs were selected as the carrier material for preparing the core–shell nanoparticles (CSs) as a kernel.

Next, thin film hydration was employed to prepare ligand-modified core–shell nanoparticles (CSs), as illustrated in Fig. 1F. Initially, Aβ11-modified CSs (Aβ11@CSs) were synthesized to screen the content of Chol-PEG2000-Aβ11 using IVIS imaging. As demonstrated in Fig. 1G, H, Additional file 1: Figure S2, Aβ11@CSs displayed a higher fluorescence intensity in the brain compared to CSs alone, indicating Aβ11’s robust targeting ability. Notably, the brain’s fluorescence signal was significantly higher than in other groups when the molar ratio of Chol-PEG2000-Aβ11 was 5%. The fluorescence intensities for 1% and 10% Aβ11 in the brain did not exhibit a significant difference. Subsequently, the study investigated whether Tween 80 (T80) could enhance the penetration of Aβ11@CSs through the blood–brain barrier (BBB). As shown in Fig. 1I, J, Additional file 1: Figure S2, the incorporation of T80 led to increased accumulation of nanoparticles in the brain compared to Aβ11@CSs. Interestingly, optimal distribution was observed with 0.5% T80 (v/v), and higher concentrations of T80 did not further enhance fluorescence intensity. This could be attributed to T80's ability to inhibit P-glycoprotein, thereby preventing nano efflux, and its potential competition with Aβ11 for binding to lipoproteins, which might reduce Aβ11's efficiency [12, 28]. Based on these findings, a composition of 5% Chol-PEG2000-Aβ11 and 0.5% T80 was selected for modifying the nanoparticles (Aβ11/T80@CSs) in subsequent studies.

### Characterization of the Aβ11/T80@CSs nanodrug delivery system

**Fig. 2:**
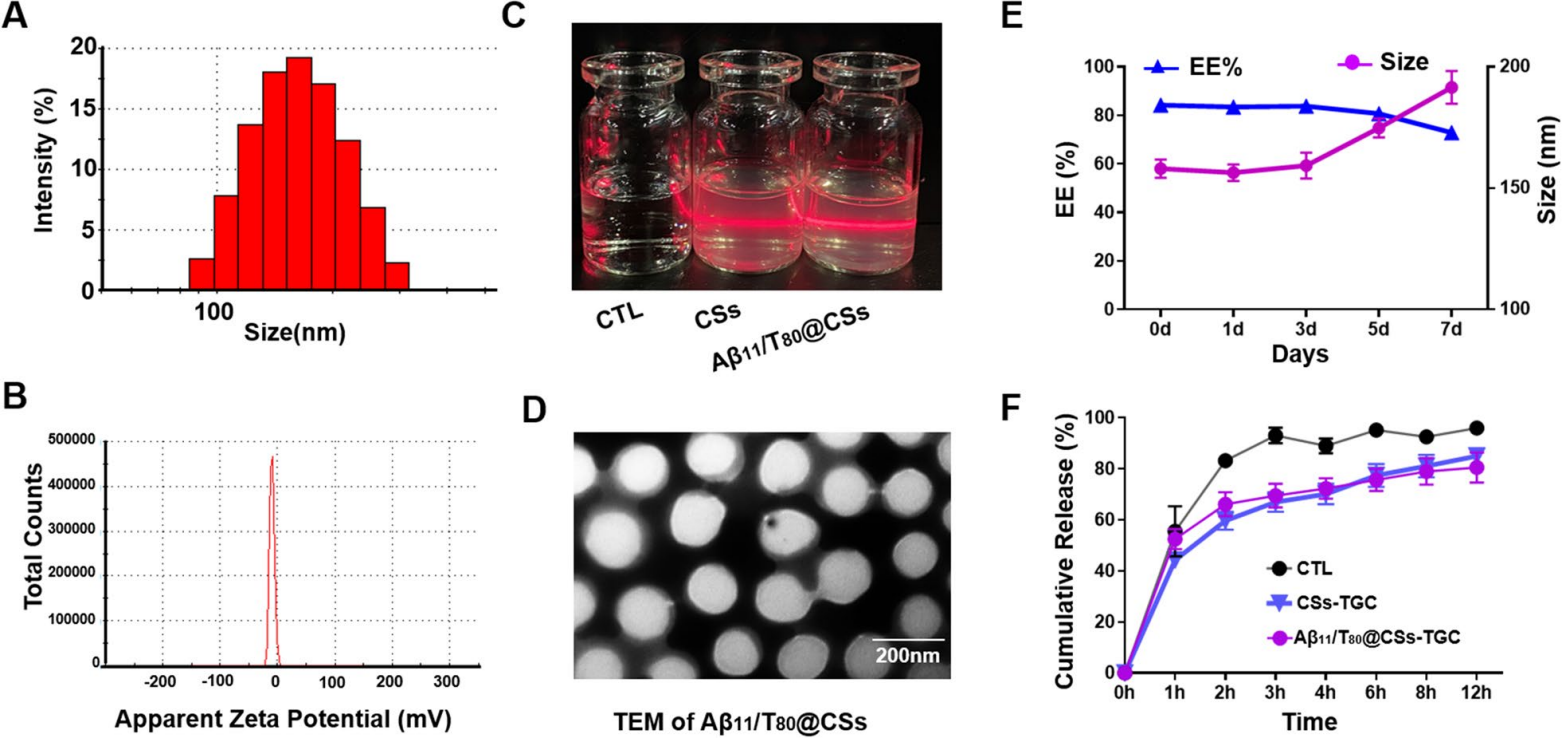
Characterization of Aβ11/T80@CSs. **A** The size distribution of Aβ11/T80@CSs was analyzed. **B** Zeta potential measurements were conducted to assess the surface charge of Aβ11/T80@CSs. **C** The Tyndall effect was employed to characterize the appearance and colloidal nature of Aβ11/T80@CSs. **D** Transmission Electron Microscopy (TEM) was used to visualize the structural morphology of Aβ11/T80@CSs. **E** The storage stability of Aβ11/T80@CSs at 4 °C was evaluated by measuring particle size and encapsulation efficiency on days 0, 1, 3, 5, and 7. **F** The release profile of Aβ11/T80@CSs in phosphate-buffered saline (PBS) was investigated using a dynamic dialysis method. All data are expressed as the mean ± standard deviation (SD), based on n = 3 independent experiments

The physical, chemical properties, and stability of Aβ11/T80@CSs were characterized. The average particle size of Aβ11/T80@CSs-TGC was found to be 158 ± 3.1 nm, accompanied by a negative zeta potential of −11.7 ± 0.6 mV (Fig. 2A, B). These findings indicated a uniform particle size distribution and a single-peak zeta potential distribution for Aβ11/T80@CSs. Notably, the particle size of Aβ11/T80@CSs was approximately 20 nm larger than that of the PLGA NPs, a difference that was not statistically significant. High-Performance Liquid Chromatography (HPLC) was utilized to ascertain the Tigecycline (TGC) content, revealing an encapsulation efficiency (EE%) of 84.2 ± 1.3% for Aβ11/T80@CSs-TGC. Under natural light, both Aβ11/T80@CSs-TGC and CSs-TGC solutions appeared clear, transparent, and light blue in color. Upon exposure to a laser pointer, aside from the control, Aβ11/T80@CSs-TGC and CSs-TGC exhibited a pronounced Tyndall effect (Fig. 2C), demonstrating that the preparation was a homogeneous colloidal solution. Transmission Electron Microscopy (TEM) images revealed a homogeneous spherical structure with a core–shell configuration for Aβ11/T80@CSs-TGC (Fig. 2D).

The stability of Aβ11/T80@CSs-TGC was evaluated by storing the nanoparticles at 4 °C for 5 days. As illustrated in Fig. 2E, there were no significant changes in particle size and EE% during this storage period, suggesting excellent stability of the formulation. The dynamic dialysis method was employed to examine the in vitro drug release profile. As depicted in Fig. 2F, approximately 50% of TGC was released from the nanoparticles within the first hour, followed by a sustained release over time. The cumulative drug release within 12 h was 85 ± 2.3% for CSs-TGC and 80.5 ± 4.8% for Aβ11/T80@CSs-TGC, indicating that ligand modification did not significantly impact the release of TGC from the nanoparticles. The complete release of the free drug within three hours could be ascribed to the absence of a carrier material for encapsulation. Overall, these results demonstrated that Aβ11/T80@CSs was highly stable and could efficiently entrap and release TGC.

### Targeting efficiency of Aβ11/T80@CSs

**Fig. 3:**
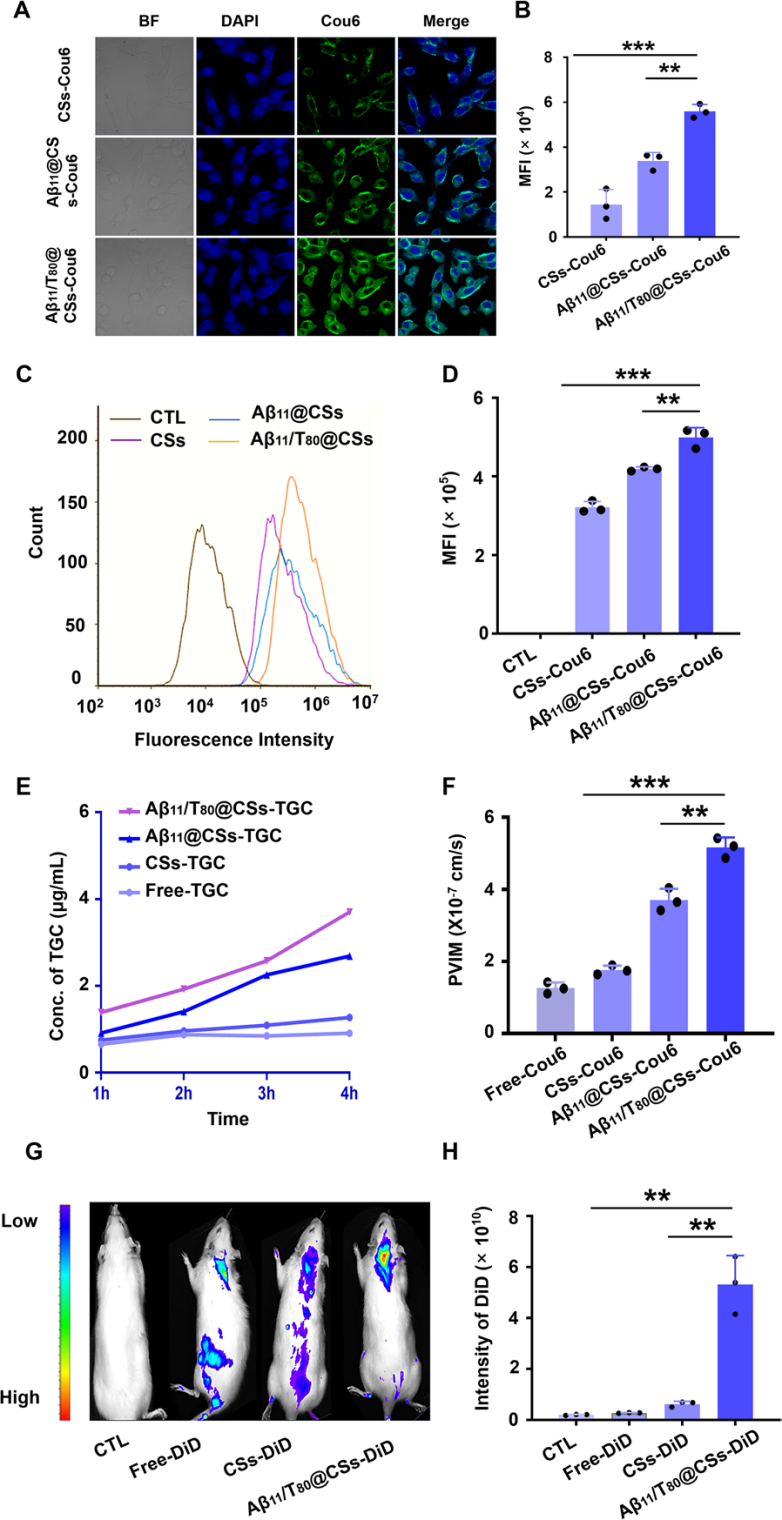
Effective uptake and brain distribution of Aβ11/T80@CSs. **A** Uptake of CSs, Aβ11@CSs, and Aβ11/T80@CSs by bEnd.3 cells was visualized using laser confocal microscopy. **B** Quantification of fluorescence intensity from the uptake study. **C** PBS, CSs, Aβ11@CSs, and Aβ11/T80@CSs were incubated with bEnd.3 cells for 4 h, followed by **D** analysis of fluorescence using flow cytometry. **E** The cumulative transport volume of nanoparticles across the blood–brain barrier (BBB) was assessed at 4 h. **F** Calculation of the transcellular membrane apparent permeability coefficient (Papp) in vitro. **G** Fluorescence imaging of brains from intracranially infected rats. **H** Quantitative analysis of brain fluorescence. All data are presented as the mean ± standard deviation (SD), based on n = 3 independent experiments. Statistical significance was determined using a t-test in panels **B**, **D**, **F**, and **H**, with **p < 0.01, ***p < 0.001, ****p < *0.0001* indicating levels of significance

Effective drug delivery across the blood–brain barrier (BBB) is crucial in the treatment of intracranial infections, as it enables access to the infection site, enhances drug efficacy, minimizes side effects, prolongs drug delivery, and potentially overcomes resistance. Strategies and technologies of the Aβ11/T80@CSs nanodrug delivery system aimed at improving BBB penetration are pivotal for advancing treatment options for intracranial infections. We investigated the uptake of different nanoparticles at the 4-h mark using confocal laser microscopy, employing bEnd.3 cells as a model system. As depicted in Fig. 3A, CSs, Aβ11@CSs, and Aβ11/T80@CSs were internalized by bEnd.3 cells. Notably, Aβ11/T80@CSs demonstrated significantly higher cellular uptake, with fluorescence intensities 3.9 times and 1.65 times greater than those of CSs and Aβ11@CSs, respectively (Fig. 3B). This finding was corroborated by flow cytometry results, which showed that the fluorescence intensity of Aβ11/T80@CSs was four times and 2.5 times higher than that of CSs and Aβ11@CSs, respectively, signifying that nanoparticle modified with Aβ11 and Tween 80 significantly augmented cellular uptake (Fig. 3C, D).

In this study, an in vitro BBB model was developed to examine the transport capacity of free Tigecycline (TGC), CSs, Aβ11@CSs, and Aβ11/T80@CSs across the endothelial barrier monolayer. As illustrated in Fig. 3E, F, free TGC exhibited the lowest permeability in monolayer bEnd.3 cells and the weakest ability to traverse the BBB. The CSs-TGC group showed limited BBB permeability within the initial four hours, with a gradual increase over the subsequent three hours. However, the dual-ligand Aβ11/T80@CSs displayed enhanced transport capacity in the in vitro BBB model compared to untargeted CSs and single-ligand Aβ11@CSs, with transshipment progressively increasing over time. Investigating the distribution of formulations in a brain infection model provided insights into the nanoparticles' ability to penetrate the BBB post-infection onset. This approach enabled a more accurate and comprehensive assessment of nanoparticle penetration capabilities, as the model more closely reflected BBB properties. The results revealed that the fluorescence intensity of Aβ11/T80@CSs-DiD was 19 times that of free DiD and 8.6 times that of CSs-DiD (Fig. 3G, H), a significant difference, indicating that the nano-delivery system possessed exceptional brain targeting capabilities.

### Pharmacokinetics and pharmacodynamic study of Aβ11/T80@CSs-TGC

**Fig. 4:**
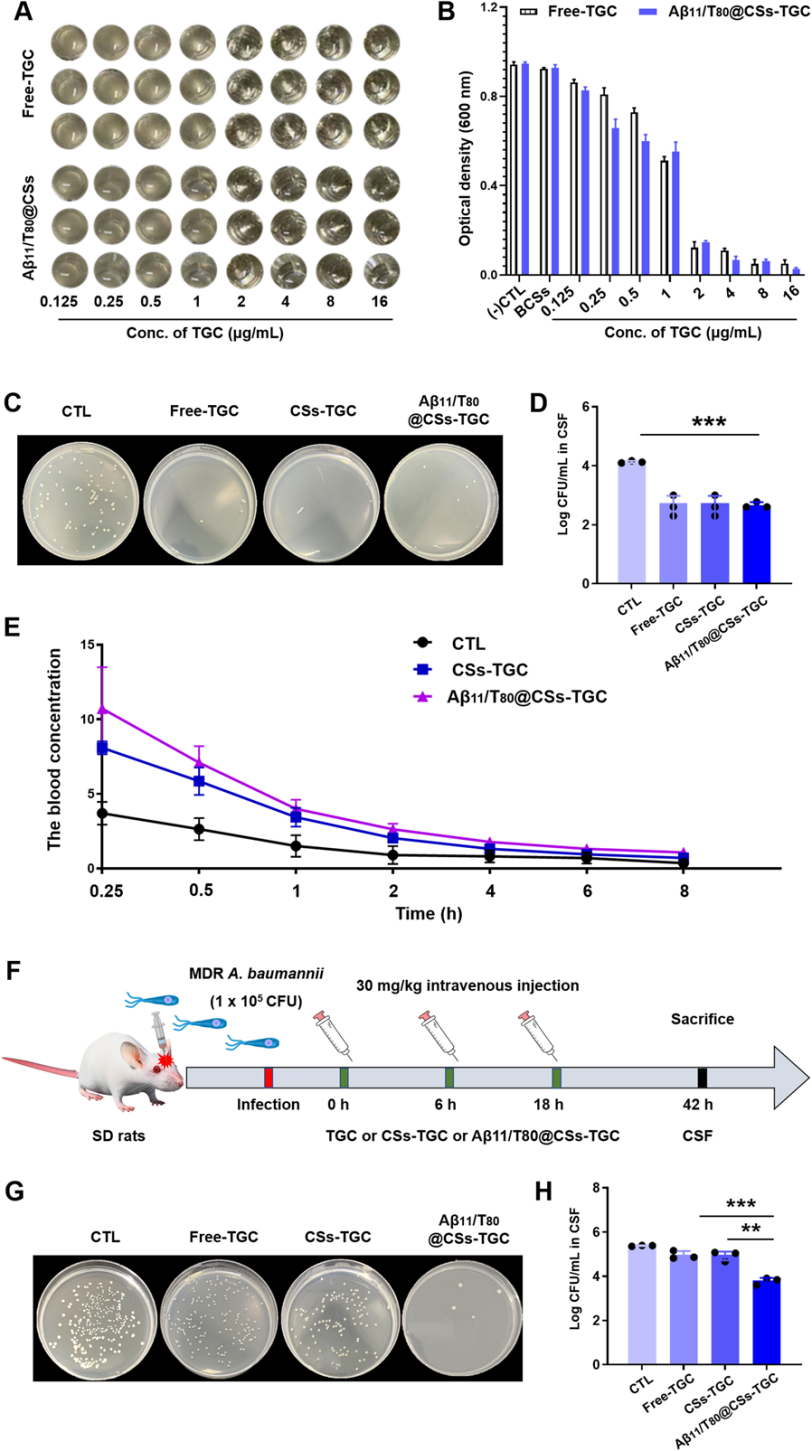
Enhanced antibacterial activity of TGC by Aβ11/T80@CSs. **A** Digital images and **B** Optical Density (OD) at 600 nm of Aβ11/T80@CSs, determined using the microbroth dilution method. **C** Digital images and **D** bacterial count statistics in rat cerebrospinal fluid (CSF) following intrathecal injection of free TGC, CSs, and Aβ11/T80@CSs. **E** Plasma concentration–time curves following intravenous administration of free TGC, CSs-TGC, and Aβ11/T80@CSs-TGC. **F** Experimental protocol for the infection and treatment of multidrug-resistant (MDR) *A. baumannii* **G** Digital images and **H** bacterial count statistics in CSF of different groups after intravenous injection. Pharmacokinetic parameters including maximum concentration (Cmax), terminal elimination half-life (T1/2), area under the curve AUC(0-t), and clearance (CL) are presented. All data are expressed as the mean ± standard deviation (SD), based on n = 3 independent experiments. Statistical significance in panels D and H was determined using a t-test, with **p < 0.01, ***p < 0.001, ****p < *0.0001* indicating levels of significance

In recent years, the prevalence of multidrug-resistant (MDR) *A. baumannii* has increased. Currently, TGC and polymyxin drugs are among the few effective treatments against infections caused by MDR *A. baumannii*. Nano-delivery systems have emerged as a promising strategy to overcome drug resistance by enhancing drug concentration, cellular uptake, and targeted delivery. These systems can bypass resistance mechanisms, deliver combination therapies, and enable programmable drug release, thereby enhancing the efficacy of therapeutic agents and contributing to improved treatment outcomes in resistance-prone conditions. We assessed the antimicrobial activity of Aβ11/T80@CSs-TGC against MDR *A. baumannii*. In vitro antibacterial experiments demonstrated that neither PBS nor blank core–shell nanoparticles inhibited bacterial growth. Both free TGC and Aβ11/T80@CSs-TGC displayed potent antimicrobial activity with a minimum inhibitory concentration of 2 μg/mL (Fig. 4A, B), indicating TGC’s effectiveness against MDR *A. baumannii*. Cerebrospinal fluid (CSF) is a clear, colorless body fluid found in the brain and spinal cord. Bacteria predominantly reside in the CSF. To explore the in vivo anti-infective efficacy of Aβ11/T80@CSs-TGC, intrathecal injections of various formulations were administered directly into the CSF, and the antibacterial efficacy of TGC was observed. As shown in Fig. 4C, D, Aβ11/T80@CSs-TGC achieved an antibacterial rate of 90% in the CSF, indicating Aβ11/T80 modification does not affect the release and anti-*A. baumannii* activity of the TGC drug in CSF.

Additionally, the study quantified the blood drug concentration of TGC at various time points following intravenous administration of nanoparticles, with the related pharmacokinetic parameters presented in Table 3. Notably, the peak concentrations of CSs and Aβ11/T80@CSs were significantly higher than those of the free TGC group (Fig. 4E). CSs-TGC and Aβ11/T80@CSs-TGC exhibited similar pharmacokinetic profiles. The area under the curve (AUC_0-8 h) for Aβ11/T80@CSs-TGC was 21.62 ± 1.79 μg/mL*h, approximately 2.5 times that of free TGC. Furthermore, the clearance (CL) of TGC was significantly reduced in nanoparticle formulations compared to free TGC. The CL for CSs-TGC and Aβ11/T80@CSs-TGC decreased by factors of 2 and 2.76, respectively. These results suggest that nanoparticle usage could prolong the blood circulation time of TGC and enhance its bioavailability.

**Table 3:** Pharmacokinetic parameters after intravenous injection in rats

Pharmacokinetic	Unit	Free-TGC	CSs-TGC	Aβ11/T80@CSs-TGC
Cmax	μg/mL	3.70 ± 0.62	8.09 ± 0.35**	10.72 ± 2.26***
Tmax	h	0.25	0.25	0.25
AUC(0-t)	μg/mL*h	8.48 ± 2.90	16.51 ± 0.09*	21.62 ± 1.79**
T1/2	h	0.77 ± 0.24	0.91 ± 0.21	1.26 ± 0.21
MRT(0-t)	h	2.6 ± 0.16	2.29 ± 0.10	2.39 ± 0.14
CL	L/h/kg	1.20 ± 0.25	0.59 ± 0.03	0.43 ± 0.02

(*x̅* ± SD, n = 3, compared with Free-TGC, ^*^p < 0.5, ^**^p < 0.01, ^***^p < 0.001)

Encouraged by these findings, we proceeded to intravenously administer physiological saline, free TGC, CSs-TGC, and Aβ11/T80@CSs-TGC to rats. The results revealed that Aβ11/T80@CSs-TGC significantly inhibited the growth of multidrug-resistant (MDR) *A. baumannii* in the cerebrospinal fluid (CSF). The colony counts in the Aβ11/T80@CSs-TGC group were markedly lower than those in the other groups (Fig. 4F–H). These outcomes suggested that the ligand-modified nanoparticles enhanced the distribution characteristics of TGC, facilitating its penetration through the blood–brain barrier (BBB) and thereby exerting effective antibacterial effects.

### Preliminary biosafety assessment of Aβ11/T80@CSs

**Fig. 5:**
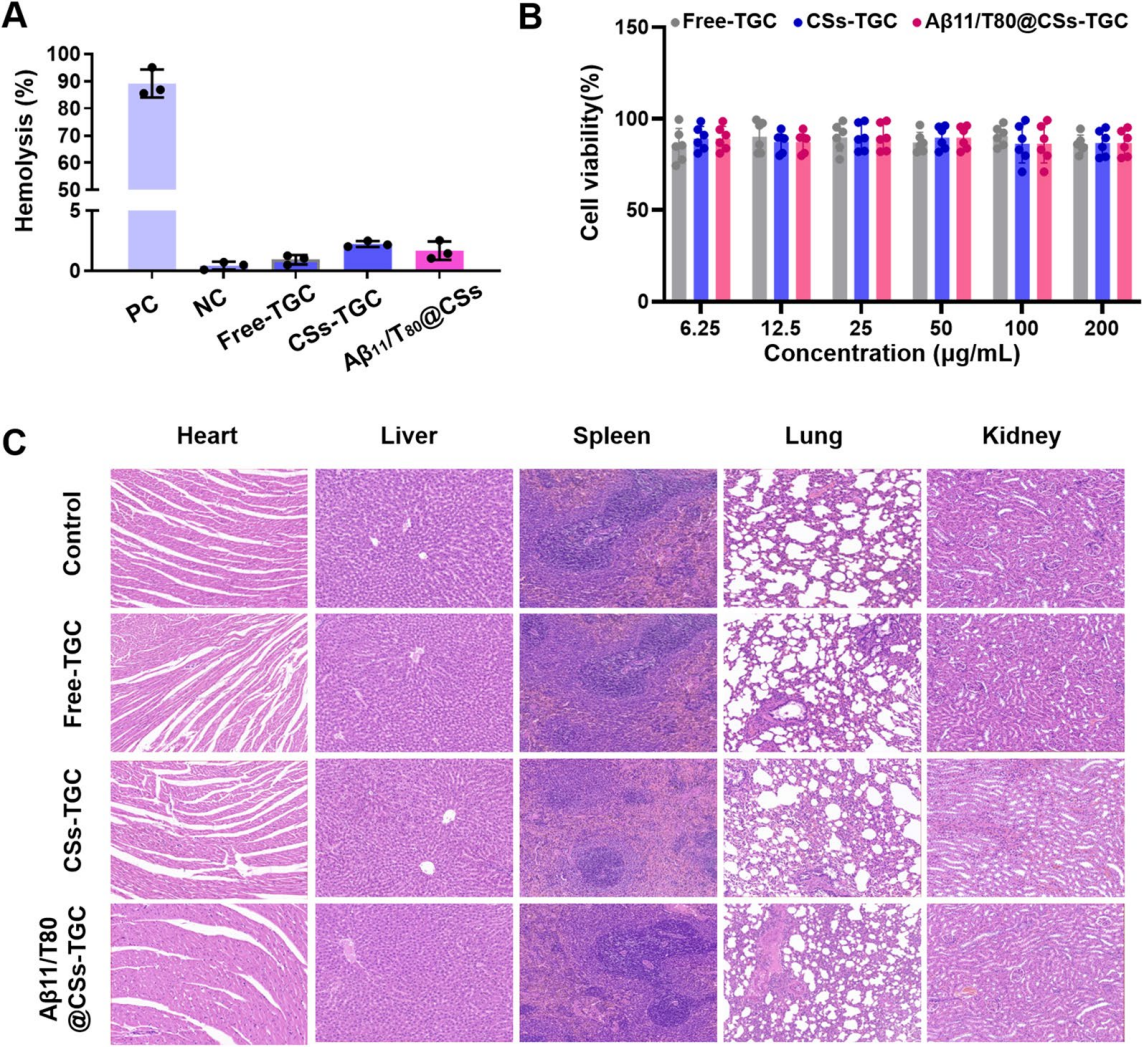
Biosafety Assessment of Aβ11/T80@CSs. This figure presents the results of our biosafety evaluation. **A** We conducted in vitro hemolysis assays on various formulations, employing pure water as the positive control (PC) and saline as the negative control (NC). **B** The viability of bEnd.3 cells was examined following a 24-h incubation with different concentrations of free TGC, CSs-TGC (**C**), and Aβ11/T80@CSs-TGC (**D**). Additionally, histopathological examinations of the heart, liver, spleen, lungs, and kidneys were performed across the different groups, two days post-treatment (Panel **E**). All data are expressed as the mean ± SD, n = 3 independent experiments. Experiment E was repeated 3 times independently

The biosafety of Aβ11/T80@CSs was assessed through the evaluation of blood biochemical indices and histopathological examination using hematoxylin and eosin (H&E) staining. As depicted in Fig. 5A, the hemolysis rates for both nano-formulations and the free TGC group were under 5%, relative to the positive control. This finding indicates that the Aβ11/T80@CSs formulation is biocompatible, exhibiting negligible hemolytic activity on erythrocytes. Additionally, the cytotoxic effects of TGC, CSs-TGC, and Aβ11/T80@CSs-TGC on bEnd.3 cells were quantified utilizing the MTT assay. Notably, cell survival rates exceeded 80% across a range of TGC concentrations (Fig. 5B). Additionally, the blood biochemistry indexes, including alanine aminotransferase (ALT), aminotransferase (AST), creatinine (CREA), and urea (UREA) (Additional file 1: Figure S1). Furthermore, histological analysis of the heart, liver, spleen, lungs, and kidneys, stained with H&E, revealed no significant pathological alterations in the TGC, CSs-TGC, and Aβ11/T80@CSs-TGC groups in comparison to the normal control group (Fig. 5C). These findings corroborate the hypothesis of the enhanced biosafety profile of Aβ11/T80@CSs-TGC both in vitro and in vivo. Consequently, these results advocate for the potential of Aβ11/T80@CSs as a clinically viable nanocarrier system for targeted brain delivery of TGC, offering therapeutic avenues for treating intracranial infections.

## Conclusion

In summary, we successfully synthesized core–shell nanoparticles, modified with Aβ11 and Tween 80, for the delivery of Tigecycline (TGC) aimed at treating intracranial infections caused by multi-drug resistant (MDR) *A. baumannii*. The Aβ11/T80@CSs nanoparticles demonstrated an effective encapsulation of the water-soluble anti-infection drug TGC in vitro, and exhibited significant activity against MDR *A. baumannii*. Crucially, the Aβ11/T80@CSs-TGC formulation effectively inhibited the growth of MDR *A. baumannii* in cerebrospinal fluid (CSF). Consistently, these findings suggest that the Aβ11/T80@CSs nano-delivery system has considerable potential to enhance the efficacy and safety of treatments for brain diseases (as depicted in abstract graphic). However, further research is imperative to evaluate the clinical applicability of this nano-delivery system and to explore its potential in the treatment of various brain disorders.

### Supplementary Information

**Additional file 1: Figure S2.** Raw data-Fig. 1H.

## Data Availability

Not applicable.
