# Supplementary material for: Dual-targeting tigecycline nanoparticles for treating intracranial infections caused by multidrug-resistant Acinetobacter baumannii
Source: J Nanobiotechnology. 2024 Mar 30;22:138. doi: 10.1186/s12951-024-02373-z (PMC10981309; doi:10.1186/s12951-024-02373-z)
Supplement: Supplementary file 1 — Additional file 1: Figure S2. Raw data-Fig. 1H. [file 12951_2024_2373_MOESM1_ESM.pdf]

Supplementary material S1

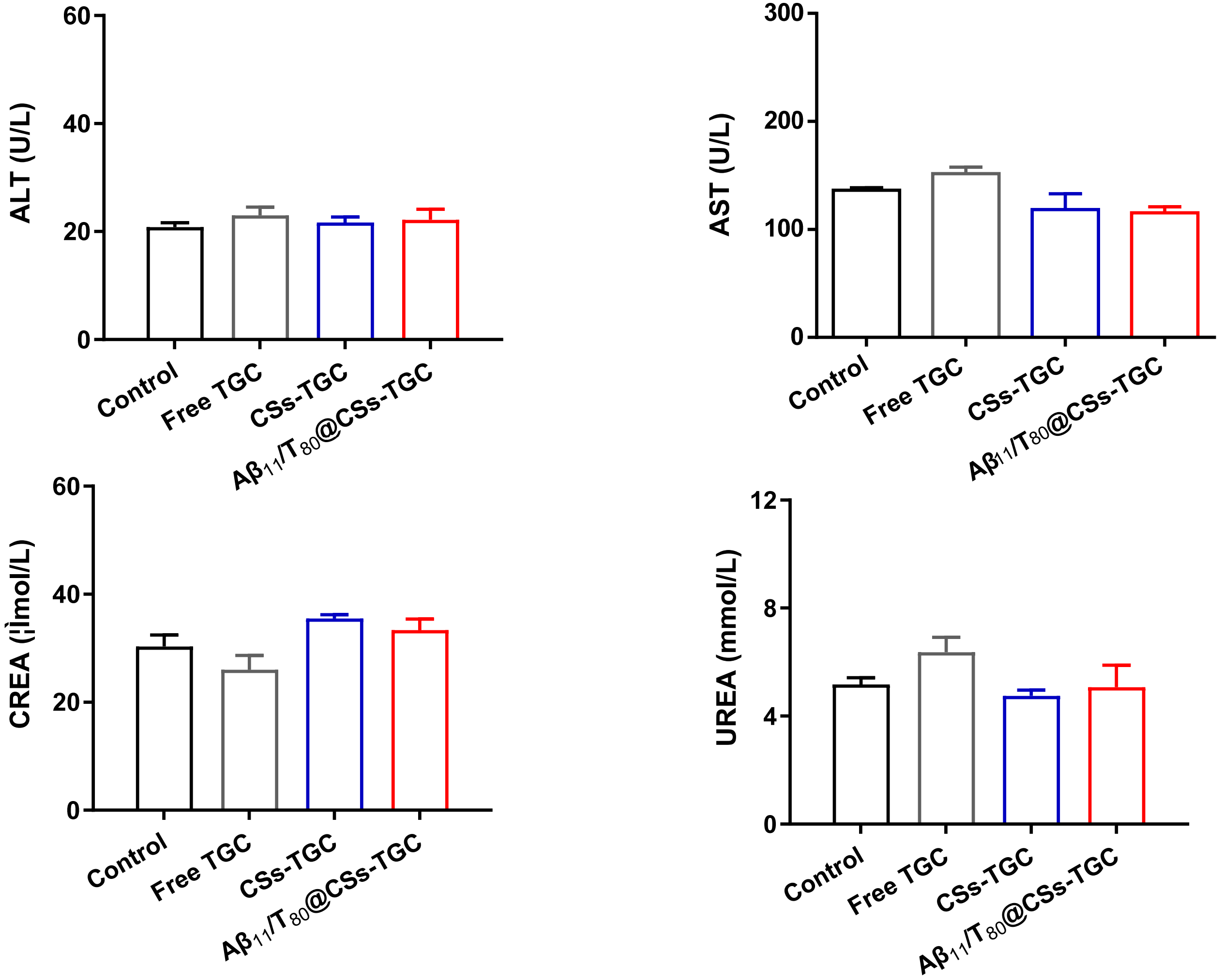

Supplementary material S2

Raw data-Figure 1H

Control			Free-DiD			CSs-DiD			1%Aβ $_{11}$			5%Aβ $_{11}$			10%Aβ $_{11}$		
2.11	1.81	2.21	2.39	2.29	2.33	3.46	3.15	3.51	5.93	5.23	6.41	7.91	7.18	7.85	6.05	6.09	6.01

Raw data-Figure 1J

Control			Free-DiD			CSs-DiD			5%Aβ $_{11}$			5%Aβ $_{11}$+0.5%T_{80}			5%Aβ $_{11}$+1%T_{80}			5%Aβ $_{11}$+1.5%T_{80}			5%Aβ $_{11}$+2%T_{80}		
2.14	1.80	2.19	2.39	2.29	2.33	3.46	3.15	3.51	7.91	7.18	7.85	9.43	11.10	9.37	9.14	8.45	8.79	9.99	8.89	8.56	9.81	8.79	9.00
